# Endothelin-1 Expression in Prostate Needle Biopsy Specimens Correlated With Aggressiveness of Prostatic Cancer

**Published:** 2017-04-08

**Authors:** Mojgan Asgari, Elham Eftekhar, Maryam Abolhasani, Hossein Shahrokh

**Affiliations:** 1 *Oncopathology Research Center, Iran University of Medical Science (IUMS), Tehran, Iran*; 2 *Sherkat Naft Hospital, Tehran, Iran*; 3 *Uro Oncology Department, Hasheminejad Kidney Center, Iran University of Medical Sciences (IUMS), Tehran, Iran*

**Keywords:** Endothelin-1, Prostatic cancer, Prognosis, Needle biopsy

## Abstract

**Background & Objective:**

As the prostate adenocarcinoma is one of the most common malignant tumors in males, looking for a marker to effectively predict aggressiveness and metastatic potential in an apparently localized cancer in initial needle biopsy specimens can help the clinicians to make more appropriate decision for treatment, planning, and choosing appropriate targeted therapy. The present study assessed the value of Endothelin-1 expression to predict prognosis of prostatic cancer

**Methods:**

In a cross sectional study, 83 patients who underwent radical prostatectomy in Hasheminejad Kidney Center in 2008 through 2012 were assigned to two groups including 43 with and 40 without extra-prostatic extension (EPE). Endothelin-1 staining was performed on Paraffin Embedded blocks of preoperative needle biopsies.

**Results:**

The expression of Endothelin-1 increased in 72% of patients in the group with EPE (P<0.001). The group with Endothelin-1 positivity showed higher serum level of prostate specific antigen (PSA) (p = 0.039). Endothelin-1 expression was positive in 67% of patients with perineurial invasion (P<0.001). Adjusting the baseline variables of PSA and PN in a multivariable logistic regression model, the Endothelin-1 positivity could effectively predict EPE in patients with prostatic cancer (OR: 5.46, p = 0.010).

**Conclusion:**

Correlation of Endothelin-1 expression in needle biopsy specimens in expected with extra-prostatic extension of tumor in radical prostatectomy specimens, perineurial invasion and serum PSA level at the time of diagnosis.

## Introduction

According to annually published National Cancer Registration Report 2008-2009 of Iran, prostate cancer is the fourth most frequent cancer among male adults with an annual cause of death in 4.5 per 100,000 men in Iran (1,2,3). As there are no integrated national programs for screening and recording of prostate cancer, the incidence rate of this cancer might be more than these registered data (2). Despite the relatively high prevalence of this cancer in our region, most patients remained asymptomatic except in advanced stages of cancer that manifested by weight loss, urinary tract obstruction and skeletal bone pains. To screen and early detect, serum level of prostate specific antigen (PSA) in combination with digital rectal exam are used for suspected patients who referred for Ultrasound-Guided Transrectal Needle Biopsy (4). Preoperative PSA level, TNM staging, Gleason grade and surgical margins are now considered as major prognostic factors for prostate carcinoma. The second line factors include tumor volume, histologic type and DNA ploidy analysis and some biologic markers including ki67, p53, and p21 (5). One of the interesting and challenging biomarkers is Endothelin-1 (ET-1). Endothelin-1 is produced by many different cell types (6), including prostatic epithelium (7) and is known as one of the most potent endogenous vasoconstrictors with prolonged effects (8,9). Elevated plasma level of this marker has been shown in patients with metastatic prostate cancer comparing the patients who are involved by organ-confined cancer as well as healthy people (10). Two receptor subtypes of endothelin-1 are Endothelin receptor A (ET_A)_ and Endothelin receptor B (ET_B_). It has been demonstrated that the binding of Endothelin-1 to ET_A_ can induce survival pathway, as well as induce cell proliferation by activating kinases such as epidermal growth factor, and inhibits apoptosis (10–11), while the activation of the ET_B_ can result in clearance of circulating Endothelin-1 as well as stimulation of apoptosis. Endothelin-1 regulates neovascularization by controlling endothelial cell migration, proliferation, and invasion as well as induction of micro-vessel density and production of vascular endothelial growth factor in tumor cells (12,13). On the other hand, Endothelin-1 inhibits apoptosis by modulation of cell survival pathways, such as PI3-K-dependent AKT activation (15). In addition, endothelin-1 is a mitogenic factor for osteoblasts whereas results in reduced osteoclastic bone resorption, osteoclast motility and therefore plays an enhancing role in osteoblastic metastases (14,15). In the few last decades, preliminary data from clinical trials on endothelin receptor antagonists such as Atrasentan in patients with prostate cancer have been encouraging; however they have not reached to a definite result as a consensus yet (17). In the present study, we aimed to investigate the association of Endothelin-1 expression by immunohistochemistry (IHC) on needle biopsies with different prognostic factors of prostatic cancer including extra-prostatic extension in radical prostatectomy specimens, serum level of PSA, and Gleason´s score of the tumor. 

## Material and Methods

All radical prostatectomy specimens prepared in Hasheminejad Kidney Center in Tehran from 2008 to 2012 were retrospectively reviewed and 83 specimens selected were assigned in two groups including 40 patients with extra-prostatic extension and 43 patients without EPE. The preoperative H&E slides of the patients’ needle biopsies were extracted and reviewed; in the present study, the paraffin blocks of needle biopsy cores which showed most involvement by tumor with highest Gleason´s score were selected. Other study parameters which assessed were patients’ age, initial serum level of PSA, Gleason´s score, perineurial invasion and adjacent organs involvement (table 1). The exclusion criteria included small amount of tissue in paraffin blocks and crushed specimens, and unavailable data on preoperative biopsy specimen or serum PSA level. Immunohistochemistry was performed using the envision method (Abcam company, USA) on formalin fixed tissue sections and pretreated with heat-induced epitope retrieval for Endothelin-1 using Anti-Endothelin- 1 antibody (TR.ET.48.5) (ab2786). Normal colonic mucosa was used as positive control and slides with omitted primary antibody as negative control.

IHC stained sections were classified in four groups in a semi-quantitative scoring system based on intensity of cytoplasmic staining in tumor cells: 0 as negative; 1 as weak cytoplasmic staining in more than half of the tumoral cells; 2 as moderate staining in more than half of tumor cells; and 3 as strong staining in more than half of tumor cells. Scores 0 and 1 were considered as low Endothelin-1 expression or negative and scores 2 and 3 as high Endothelin-1 expression or positive (15,16). 

Results were presented as mean ± standard deviation (SD) for quantitative variables and were summarized by absolute frequencies and percentages for categorical variables. Categorical variables were compared using chi-square test or Fisher's exact test when more than 20% of cells with expected count of less than 5 were observed. Quantitative variables were also compared with Mann- Whitney U test. Multivariable logistic regression analysis was used to assess the value of Endothelin-1 to predict tumor-related outcome. Statistical significance was determined as a p value of ≤ 0.05. All statistical analysis was performed using SPSS software (version 16.0, SPSS Inc., Chicago, Illinois). 

**Table 1:** Frequency and Percentage of Pathology Findings and the Result of staining

Variable		Frequency (%)
Gleason´s score	6	28(33.7%)
	7	33(39.8%)
	8	17(20.5%)
	9	4(4.8%)
	10	1(1.2%)
Extraprostatic Extension	Positive	43(51.8%)
	Negative	40(48.2%)
Perineurial invasion	Positive	43(51.8%)
	Negative	40(48.2%)
IHC Score for ET-1	0	19(22.9%)
	1	24(28.9%)
	2	26(31.3%)
	3	14(16.9%)

**Fig 1:**
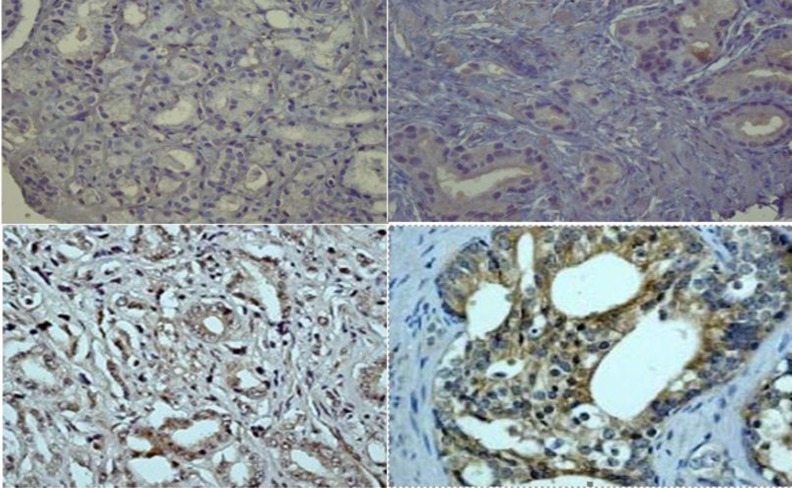
a) Score 0; no immunoreactivity in tumor cells b) Score 1; weak cytopasmic staining in more than half of tumor cells. c) Score 2; moderate cytopasmic staining in more than half of tumor cells. d) Score 3; severe cytopasmic staining in more than half of tumor cells.

## Results

Pathologic findings and the result of staining are summarized in Table 1. There was no difference in mean age of patients in the groups with and without positive Endothelin-1 (65.6 ± 7.4 years versus 64.5 ± 8.3 years, *p*=0.533). Of 43 patients without EPE, 26.0% had positive Endothelin-1 expression, while 72.0% of those with EPE have shown to be Endothelin-1 positive with a significant difference (*P*<0.001). Forty three cases had perineurial invasion including 33.0% in the group with negative Endothelin-1 expression and 67% in those with positive Endothelin-1 expression. In this regard, 73% of patients without perineurial invasion were negative for Endothelin-1. The patients group with positive Endothelin-1 expression had a mean serum PSA level of 44.6 ± 10.2 ng/ml while, the mean serum level of this marker in the group with negative Endothelin-1 expression was 18.6 ± 6.7 ng/ml with a significant difference (P=0.039). In total, 28 patients showed Gleason’s score 6 that of them, 32.1% were positive for Endothelin-1. Overall, Endothelin-1 positivity was shown in 54.5% of patients with Gleason score 7, in 58.8% of patients with Gleason score 8, in 50.0% of patients with Gleason score 9, and also in 100% of those with Gleason score 10 with no inter-group difference (*p*=0.327). Multivariate logistic regression analysis was performed to assess the role of positive Endothelin-1 to predict EPE. Adjusting the baseline variables of PSA and PN, the Endothelin-1 positivity could effectively predict EPE in patients with prostatic cancer (OR: 6.04, *p*= 0.010). The result of were summarized in table 2.

**Table 2:** ET-1 Expression Regarding Patient’s Age, Serum PSA and Pathologic Findings.

Variable	Group	ET-1 positive	ET-1 negative	P value	OR (95%CI)
Age	-	64.5 ± 8.3	65.6 ± 7.4	0.533	-
PSA, ng/ml	-	18.6 ± 6.7	44.6 ± 10.2	<0.001	-
Perineurial Invasion	negative	11(27.5%)	29(72.5%)	<0.001	5.46 (1.41 – 14.10)
	positive	29(67.4%)	14(32.6%)		
Gleason Score	6	9(32.1%)	19(67.6%)	0.261	
	7	18(54.5%)	15(45.5%)		
	8	10(58.8%)	7(41.2%)		
	9	2(50%)	2(50%)		
	10	1(100%)	0		
Extraprostatic Extension	negative	11(25.6%)	32(74.4%)	<0.001	7.67 (1.58 – 20.30)
	positive	29(72.5%)	11(27.5%)		

## Discussion

As the first available specimen of prostate cancers are needle biopsy specimens, choosing the appropriate therapeutic plan is mainly based on pathologic findings in small representatives of prostate cancer. So it is important to use all informative markers in pathology specimens to predict tumor behavior even when the examined tissue specimen is too small. A complete pathology report considering all prognostic markers would result in better handling of cancer; and using different alternatives for radical prostatectomy including radiotherapy or targeted therapy with less morbidity risk especially in patients with metastatic cancer. In recent years, several biomarkers associated with prostate cancer were examined such as endothelin-1. This marker has been identified as a promoter of tumor angiogenesis and cell proliferation and is a challenging in recent advances of targeted therapy for several human malignancies including malignant melanoma, breast and cervical carcinoma (17). It was firstly isolated and characterized as a new peptide produced by porcine aortic endothelial cells by a graduate student named Masashi Yanagisawa (8). In 1995, Nelson and his colleagues showed significant elevation of immunoreactive endothelin-1 concentration in men with metastatic prostate cancer. They showed that human prostate cancer cell line produces endothelin-1 messenger RNA and secretes immunoreactive endothelin-1. They selected 14 patients with confined prostate cancers and 16 patients with metastatic cancer and found endothelin-1 positivity in 87% of patients (10). Functions of endithelins and their receptors referred as endothelin axis in the growth and progression of various tumors trigger the clinical trials of endothelin receptor antagonists such an atrasentan in prostate cancer and the results was encouraging (20). 

In our study, we attempted to find a diagnostic tool to identify apparently localized aggressive prostate cancers in needle biopsy specimens. Our findings showed that endothelin-1was more expressed in tumoral cells of prostate cancer with EPE (72%) compared to the cancer specimens without EPE (26%). There was also a correlation between serum PSA levels and expression of ET-1 in tumor cells. Endothelin-1 was also more positive in the presence of perineurial invasion comparing the specimens without it (67% versus 27% respectively), but logistic regression analysis showed that the correlation of PSA level and perineurial invasion was dependent to the presence of EPE. We found no association between Endothelin-1 expression and patients’ age or tumor Gleason score. In 2007, Gorada et al assessed 120 cases of prostate cancer and found immunoreactivity to endothelin-1 in 72% of patients as well as a positive relationship between endothelin-1 expression and PSA levels but not with tumor Gleason score. They also concluded that high endothelin-1 expression was correlated with increased pathologic stage and tumor recurrence (16). In 2010, Menard et al selected 94 patients with prostate adenocarcinoma and reported endothelin-1 positivity in 50% of them. They found a correlation between endothelin-1 expression, PSA higher than 10ng/ml and Gleason score higher than 7 with EPE (15). Our results were supportive of Menard & Gorada study about presence of correlation between endothelin-1 expression and PSA levels and EPE. With regard to Gleason score, our results were similar to the report by Gorada, but in contrary to the results pointed by Menard. Correlation between endothelin-1 expression and perineurial invasion was not assessed by them. The study by Prez et al in 2010 performed on 68 radical prostatectomy specimens that showed higher intensity of endothelin-1 expression in specimens with positive EPE. They concluded that prostate cancer biopsy expression was an independent prognostic factor of high extracapsular stage (21). Another study by Montironi et al in 2007 showed that endothelin-1 expression was not limited to the late prostate cancer phases and was also seen in HGPIN found in radical prostatecromy and also clinically insignificant prostate cancers in cystoprostatectomy specimen; however the number of their cases was limited in each groups (22). In total, it seems that the contradictory results in the studies may be due to the difference in staining or heterogeneous nature of the tumor and the return of racial differences that should be more investigated. 

## Conclusion

Considering these results, we suggest using endothelin-1 expression as a complementary option in assessment and report of prostate needle biopsies in prostate adenocarcinoma to help the physician to make the best decision for affected patients, decrease mortality and morbidity by preventing less appropriate radical prostatectomy in aggressive tumors and also selecting patients for endothelin-1 blockade therapy. 
